# DMARD non-use in low-income, elderly rheumatoid arthritis patients: results of 86 structured interviews

**DOI:** 10.1186/ar4459

**Published:** 2014-01-29

**Authors:** Erika M Brown, Katie L Garneau, Hsun Tsao, Daniel H Solomon

**Affiliations:** 1 Division of Rheumatology, Immunology and Allergy, Brigham and Women’s Hospital, 75 Francis Street, Boston, MA 02115, USA; 2 Division of Pharmacoepidemiology and Pharmacoeconomics, Brigham and Women’s Hospital, 75 Francis Street, Boston, MA 02115, USA

## Abstract

**Introduction:**

Disease-modifying antirheumatic drugs (DMARDs) have become the treatment standard for patients with rheumatoid arthritis (RA). Although several general-population studies document that a large population of patients diagnosed with RA do not use DMARDs, little is known about this group. We explored the characteristics, experiences, and knowledge of a low-income, elderly RA population not currently using DMARDs, or receiving care from a rheumatologist.

**Methods:**

We administered structured telephone interviews to participants enrolled in a large pharmacy benefits program for the elderly who had two diagnoses of RA ≥7 days apart and no DMARD prescriptions or rheumatologist visits in the prior year. The interview contained questions concerning each participant’s sociodemographic information, disease activity, DMARD experiences, and the Modified Health Assessment Questionnaire (MHAQ). We described responses and compared prior users with never users.

**Results:**

A total of 86 people completed the interview. The mean age was 80 years and 89% were female. On average, disease duration was 20 years. Mean MHAQ score was 0.55 (SD = 0.55). Of 86 participants, 19 had previously used DMARDs, 10 of whom discontinued them because of side effects or safety concerns. Among 67 never-users, 35 (52.2%) reported that their physicians had never offered them DMARDs, 13 (19.4%) described fear of side effects, and 49 (73.1%) knew nothing about them. Prior-users reported experiencing more-severe RA symptoms than never-users.

**Conclusions:**

We found that side effects or safety concerns were the primary cause for DMARD cessation among prior-users. Among never-users, most reported never discussing or being offered DMARDs, suggesting that an educational gap may deter patients with RA from using them.

## Introduction

Rheumatoid arthritis (RA) is a chronic systemic autoimmune inflammatory disorder that increases the risk of disability and early mortality [1]. The disease manifests itself primarily in joint damage through synovial inflammation but has been shown to negatively impact respiratory, cardiac, neurologic, and hematologic systems as well [2]. It is estimated to affect between 0.5% and 1.0% of the adult population worldwide, with a higher prevalence among women and the elderly [1]. Some studies demonstrate reduced life span in RA [3-6].

Disease-modifying antirheumatic drugs (DMARDs) have been recommended as the standard of care because of their consistent reduction of pain and disability in patients that have lived RA for several decades [7]. Studies demonstrate that delays in the implementation of DMARD treatment have been associated with increased physical disability, radiologic damage, and other long-term health outcomes [8,9]. As a result, the American College of Rheumatology (ACR) and European League Against Rheumatism both recommend early and aggressive DMARD treatment to essentially all patients with RA [10,11].

Despite these recommendations, DMARD treatment rates among RA patients are suboptimal. Investigators using 4 years of Medicare and Medicaid Healthcare Effectiveness Data and Information Set data demonstrated that only 63% of RA patients used DMARDs, and use was 30% less likely among patients older than 85 years [12]. Several other insurance claims-based studies have confirmed that increasing age may act as a deterrent for using DMARDs [13-15]. Low-socioeconomic status and lack of rheumatic disease specialty care have also been identified as predictors of suboptimal DMARD use in populations, after adjusting for health care and drug-insurance benefits [15,16].

Although rates of DMARD use have been found to be low in multiple studies, we are not aware of any prior interview studies examining why certain populations do not use DMARDs. Much of the literature that currently exists comes from administrative claims data, which intrinsically lack insight into why individuals stopped or never started a DMARD regimen. This study aims to fill the current gaps in our understanding of the experiences, perceptions, and knowledge among a population of RA patients that has never used or stopped using DMARDs.

## Methods

### Participants

We performed structured telephone interviews with 86 patients with RA to better to understand the determinants and barriers to DMARD use in a low-income, elderly, and primarily female sample population. We obtained eligible subjects’ contact information from the Pharmaceutical Assistance Contract for the Elderly (PACE) in Pennsylvania, a program that provides assistance for individuals who are older than 65 years and have an annual income of less than $17,000. The PACE program supplied us with the contact information of individuals that had received a minimum of two RA diagnoses and had no prescription for DMARDs in the prior year.

The inclusion criteria for participation were eligibility for the PACE program, two physician visits coded with of a diagnosis of RA at least 7 days apart, a subject’s confirmation of his or her RA diagnosis, no prescriptions for DMARDs in the prior 12 months, and no rheumatologist visits. We excluded patients with recent rheumatology visits because one of our goals was to understand better why patients who see primary care providers for their RA do not use DMARDs.

We received ethical approval from the Partners Human Research Committee to carry out each aspect of this project, and all participants gave informed consent before their interviews.

### Interview and recruitment

We chose a two-phase system for recruitment and used US mail as our first point of contact. Subjects who did not opt out by mail were contacted by telephone. A $5 gift card was provided to individuals that completed the interview.

The interview consisted of items regarding RA, participants’ prior DMARD use, knowledge, and experience with DMARDs, the Modified Health Assessment Questionnaire (MHAQ), and subject’s sociodemographic information. We asked participants to self-report the frequency and severity of their joint stiffness, swelling by joint location (wrist, finger, elbow, knee), whether they had a blood test for RA, and if so, if it was positive or negative, and if they had nodules or bumps around their skin. To assess DMARD use, we read subjects a list of 12 common medications (generic and manufactured names) and asked if they had used one or multiple DMARDs in the past. Knowledge was determined by listing the names of DMARDs and asking participants open-ended questions regarding how much they knew about the routes of administration, side effects, benefits, costs, and drug interactions of each one. We asked never-users why they never started a DMARD regimen and prior-users why they ceased DMARD use. We also inquired whether prior DMARD users would consider using a different drug for their RA.

### Statistical analysis

Analyses were descriptive and did not test specific hypotheses. Qualitative responses to the open-ended structured interview questions were grouped into categories to facilitate analysis; quantitative responses were described by using means with standard deviations (SD) and counts. The group of respondents reporting prior use of DMARDs was compared with the never-users by using χ^2^ or Student *t* tests. We examined the specific DMARDs used by prior-users and reasons for discontinuation. Finally, the never-users’ responses to questions regarding attitudes and knowledge about DMARDs were examined. All analyses were conducted by using SAS 9.2 (Cary, NC, USA).

## Results

We sent 1,586 invitations to all eligible PACE participants via US mail. Of these, 162 (10.2%) participants chose to opt out, and 312 (19.7%) could not be delivered. We attempted telephone contact with the remaining 1,112 (70.1%), of whom 321 (20.2%) could not be reached because of a disconnected telephone line; 178 (11.2%) did not pick up the telephone; 156 (9.8%) were reported deceased; 14 (0.9%) could not speak English; 116 (7.3%) denied RA; 49 (3.1%) had seen a rheumatologist with the past year; and 2 (0.1%) reported using DMARDs.

**Figure 1:**
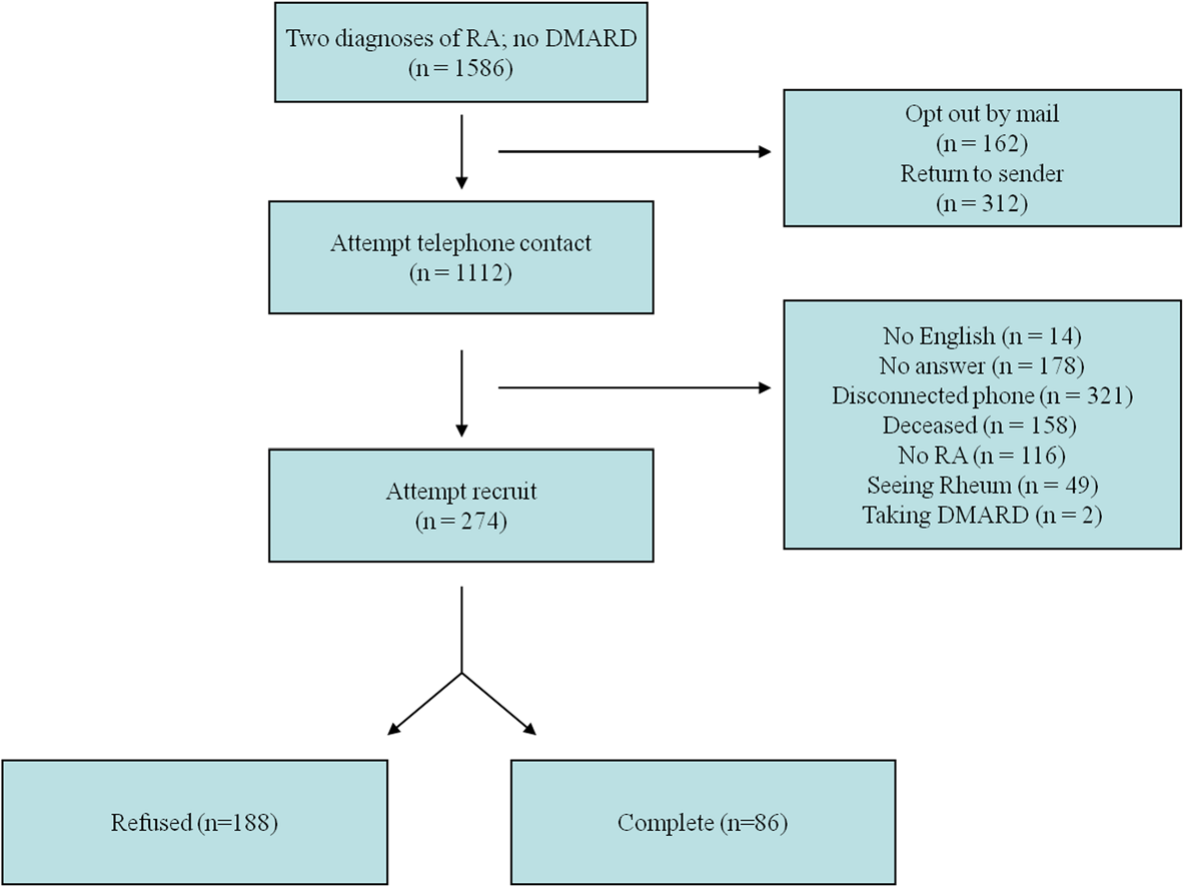
**Assembly of the study cohort.** RA, rheumatoid arthritis; DMARD, disease-modifying antirheumatic drug.

Of the remaining 274 eligible subjects, 188 of them refused to participate. Detailed information about this cohort assembly can be found in Figure 1. Our final study cohort consisted of 86 participants.

Sociodemographic information and RA-specific characteristics of our sample can be found in Table 1. The mean age of participants was 80 years; they were primarily women (89.5%) that held a high-school diploma (51.2%), and had an annual income of less than $25,000 (95.3%). On average, participants had been living with RA for 20 years; 73.3% of participants felt joint stiffness in the morning, and 61.6% self reported feeling two or more swollen joints. Only 45.3% reported having ever seen a rheumatologist. The mean MHAQ score was 0.6 (SD, 0.6).

**Table 1:** Characteristics of study population

	**Total**	**Prior-users**	**Never-users**	***P *** **value**
	***N *** **= 86 (100%)**	***n *** **= 19 (19%)**	***n *** **= 67 (81%)**	
Demographics				
Age (Mean, SD)	80 (±10.3)	79 (±5.2)	80 (±11.3)	0.77
Female	77 (89.5%)	15 (78.9%)	62 (92.5%) (Mean, SD)	0.09
Race				0.64
White, non-Hispanic	83 (96.5%)	16 (94.7%)	64 (95.5%)	
Black, non-Hispanic	3 (3.5%)	1 (5.3%)	2 (3.0%)	
Education				0.77
Less than 8th grade	10 (11.6%)	3 (15.8%)	7 (10.4%)	
Some high school	15 (17.4%)	4 (21.1%)	11 (16.4%)	
High school graduate	44 (51.2%)	8 (42.1%)	36 (53.7%)	
Some college or greater	15 (17.4%)	4 (21.1%)	11 (16.4%)	
Unknown	2 (2.3%)	0 (0.0%)	2 (3.0%)	
Income				0.59
Below $25,000	82 (95.3%)	19 (100%)	63 (94.0%)	
Above $25,000	1 (1.2%)	0 (0.0%)	1 (1.5%)	
Unknown	3 (3.5%)	0 (0.0%)	3 (4.5%)	
RA characteristics				
Morning stiffness	63 (73.3%)	17 (89.5%)	46 (68.7%)	0.07
Nodules around elbow/ankle	13 (15.1%)	3 (15.8%)	10 (14.9%)	0.93
Self-reported swollen joints				0.06
Zero	28 (32.6%)	4 (21.1%)	24 (34.8%)	
One	5 (5.8%)	1 (5.3%)	4 (6.0%)	
Two	21 (24.4%)	3 (15.8%)	18 (26.9%)	
Three	10 (11.6%)	3 (15.8%)	7 (10.4%)	
Four or more	22 (25.6%)	8 (42.1%)	15 (20.9%)	
Self-reported serologic status	50 (58.1%)	16 (84.2%)	34 (50.7%)	0.06
Positive	21 (24.4%)	10 (52.6%)	11 (16.4%)	
Negative	2 (2.3%)	1 (5.3%)	1 (1.5%)	
Do not know	25 (29.1%)	4 (21.1%)	21 (31.3%)	
Years with RA (mean, SD)	20 ± 15.9	25 ± 17.6	18 ±15.2	0.13
MHAQ (mean, SD)	0.5 ± 0.5	0.8 ± 0.6	0.5 ± 0.5	0.02
0	18 (20.9%)	1 (5.3%)	19 (28.4%)	0.01
>0-1	53 (61.6%)	12 (63.2%)	41 (61.2%)	
>1-2	11 (12.8%)	5 (26.3%)	6 (9.0%)	
>2-3	2 (2.3%)	1 (5.3%)	1 (1.5%)	
Prior visit with rheum >1 year ago	39 (45.3%)	15 (78.9%)	27 (35.8%)	0.01

MHAQ, Modified Health Assessment Questionnaire; RA, rheumatoid arthritis; SD, standard deviation.

Nineteen participants reported prior DMARD use. We report the specific drugs they discontinued and reasons i for stopping in Table 2. Five participants had previously used more than one DMARD. The majority of participants (n = 10, or 52.6%) had used methotrexate, of whom half stopped because of side effects. Approximately one-third had (n = 6) used hydroxychloroquine, 33.3% (n = 2) discontinued the drug per physician’s request, and 33.3% (two) because of inefficacy. Twenty-one percent of prior DMARD users (n = 4) had used gold.

**Table 2:** Reasons for DMARD discontinuation among 19 prior-users

	***N *** **(%)**	**Side effects experienced**	**Physician’s request**	**Cost**	**Concerns**^**a**^	**Inconvenient**	**Ineffective**	**Other**^**b**^
Methotrexate	10 (52.6)	5	1	2	1	1	2	0
Hydroxychloroquine	6 (31.6)	1	2	0	1	0	2	1
Abatacept	1 (5.3)	0	0	0	0	0	1	0
Etanercept	2 (10.5)	0	0	0	0	0	1	1
Infliximab	1 (5.3)	0	0	0	0	0	1	0
Gold	4 (21.1)	1	1	0	1	0	1	1
Total^c^	24	7	4	2	3	1	8	3

^a^Concerns about possible side effects or drug reactions that were not already experienced.

^b^Other health problems.

^c^Participants were allowed to report more than one DMARD discontinuation.

Side effects and concerns about side effects accounted for 52.6% of cessations, and inefficacy comprised 42.1%. Physicians’ requests to terminate DMARD therapy resulted in 21.1% of discontinuations, cost accounted for 10.5%, and inconvenience only made up 5.3%. However, when asked about knowledge relating to the side effects, costs, benefits, method of administration, or other aspects of specific DMARDs, few of the nonusers demonstrated any accurate knowledge.

Prior DMARD users reported more-severe RA symptoms and longer disease duration than did never-users. They had a significantly higher range of MHAQ scores than did never-users (P <0.05). Approximately two thirds of never-users reported joint stiffness in the morning, whereas nearly 90% of prior-users did (P = 0.07). Although 42.1% of prior-users reported four or more swollen joints, only 20.9% of never-users reported experiencing the same severity (P = 0.06). Prior-users reported living with RA for an average of 25 years, and never-users reported an average of 18 years (P = 0.13). Participants that had previously used DMARDs also reported more specialized services; 84.2% indicated that they had serologic blood work done, whereas only 50.7% of never-users did (P = 0.06), and 78.9% stated that they had seen a rheumatologist, whereas 35.8% of never-users did (P < 0.01).

We were also interested in the knowledge and attitudes of DMARDs among never-users (Table 3). When asked why they hadn’t started a DMARD regimen, 53.8% of never-users stated they had never been offered DMARD treatment. As well, 26.2% said they were unfamiliar with DMARDs. In response to reasons that they would consider using a DMARD, 18.5% of never-users said they would use them to relieve pain or swelling, and 12.3% said they would use them in accordance with a physician’s recommendation. One participant indicated that she would use them to stop joint damage. Only 13.8% had spoken with a physician about using DMARDs, and one individual reported researching DMARDs on her own. However, the vast majority of never-users stated they knew nothing about DMARDs.

**Table 3:** Attitudes and knowledge of DMARDs among 65 never-users

	**Never-users**
What are some reasons you never started DMARDs?	
Never heard of DMARDs	17 (26.2%)
Never been offered DMARDs	35 (53.8%)
Do not need DMARDs	10 (15.4%)
Afraid of DMARRD side effects	13 (20.0%)
Taking too many other medications	8 (12.3%)
Do not see physicians often	1 (1.5%)
Other medical problems take precedence	2 (3.1%)
Inconvenience	1 (1.5%)
What are some reasons you would use a DMARD?	
Stops joint damage	1 (1.5%)
Relieves pain or swelling	12 (18.5%)
Physician recommendation	8 (12.3%)
If rheumatoid arthritis worsens	1 (1.5%)
If the DMARD has no side effects	1 (1.5%)
Have you ever talked to a physician about any DMARDs?	
Yes	9 (13.8%)
No	56 (86.2%)
Have you ever looked up information about DMARDs?	
Yes	1 (1.5%)
No	64 (98.5%)
Do you know anything about DMARDs in general?	
Side effects	11 (16.9%)
Benefits	1 (1.5%)
Method of administration	1 (1.5%)
Nothing	53 (81.5%)
Total missing	2

Of the 67 never–users, 65 answered these questions.

## Discussion

We studied a cohort of older RA patients who had not filled prescriptions or received infusions for DMARDs in the prior year. Our findings represent data from a sample of primarily elderly, low-income women who receive insurance coverage but are not actively followed up by rheumatologists. Although several general-population studies document that a large population of patients diagnosed with RA do not use DMARDs, they do not examine the rationale behind nonuse.

To better understand the reasons for DMARD nonuse, we conducted structured interviews by speaking directly with a group of non-users’ about their attitudes, knowledge, and experience regarding the medications. We spoke with 86 participants, and found that among 19 prior-users, side effects or safety concerns were the primary causes for DMARD cessation. Prior-users also described more-severe RA symptoms than did never-users. Among 67 never-users, most reported never discussing or being offered DMARDs, suggesting that an educational gap may deter patients with RA from starting a DMARD regimen.

Our findings must be interpreted with some limitations in mind. First, we conducted structured interviews with a fairly small cohort, which affects its generalizability. The study interviews were 20 to 25 minutes in length, hindering recruitment, but allowing us to collect a larger amount of detailed data from a smaller sample.

Second, our participants were a homogeneous group of low-income, older non-users that (a) had drug insurance, and (b) had not seen a rheumatologist recently; these may limit the generalizability of their responses to the factors RA population. However, low-income, elderly individuals with RA are a group known from other studies to be at high risk for not using DMARDs [13-15], and their rationale for non-use is important. The fact that they all have insurance reduced cost as a factor for non-users.

Third, we cannot be absolutely certain that our participants had RA. We did our best to mitigate this issue by limiting recruitment to patients with two physician-reported RA diagnoses in their claims records and by having participants confirm the physicians’ diagnosis. Despite reporting no DMARD use, the study population had relatively preserved functional status, as evidence by a mean modified HAQ score of 0.5. It is likely that many of our subjects had mild RA; it is even possible that some would not fulfill criteria for RA, despite having a diagnosis from a doctor and reporting joint symptoms. According to several sets of guidelines [10,11], they would most likely benefit from DMARDs. It is possible that the study population, many of them anti-CCP negative and RF negative, may be less likely to be adherent with DMARDs. Contraindications to DMARD use may have also deterred them from using these agents.

Finally, is important to acknowledge that inaccurate information may have been reported by prior- and never–users, given that the mean disease duration of this sample is 20 years, and subjects may not recall or recognize using or learning about certain drugs.

However, it is also likely that a large portion of the reported knowledge gap came from suboptimal patient education. In a 2011 study, investigators found that primary care physicians felt uncomfortable managing DMARDs, and just over half knew when to prescribe them [17]. Given that many people living with RA in the United States do not actively see a rheumatologist, and several studies have demonstrated that patients’ personal beliefs and education about the necessity of their medication have been shown to affect greatly the courses of their medication [18,19], it is important that primary care providers be knowledgeable about the disease and its treatment. They should be able to discuss medication in a framework understood by the patients to ensure that they are making optimal decisions. The 26.2% of never-users stating they had never heard of DMARDs and 53.8% stating they had never been offered them would have likely benefited from such a conversation with a provider.

An expansion of education among nonspecialists would enable providers to feel more comfortable discussing DMARDs and provide better information to patients so they could make well-informed treatment-related decisions. More never-users would likely start using DMARDs, and non-users that would benefit from DMARD therapy would have a higher chance of reengaging as well. Despite reporting experiencing active symptoms of RA, only five of the 19 patients from this sample had tried using more than one DMARD, despite the ACR recommendation for patients with RA to try switching or using combination therapy [10].

Improving access to rheumatic disease specialists would more directly improve the probability that patients experiencing RA symptoms would use DMARDs. In 2007, a study by Deal *et al.*[20] showed that the incidence of rheumatologic and musculoskeletal diseases started to exceed the supply of rheumatologists in 2005 and predicts it will continue to do so through 2025. As such, the rheumatology community should start considering solutions to this pressing issue. One proposition is to expand the roles of mid-level practitioners such as nurse practitioners and physician assistants in rheumatology practices to enhance practice efficiency and increase the availability of less-complex services to patients [20]. We believe that mid-level practitioners’ ability to prescribe and monitor DMARD use will allow quicker and more-efficient drug use, ideally producing less need for long-term management by rheumatologists. Socioeconomic status, geographic location and access to care, comorbidities, patients’ knowledge and beliefs, physicians’ behaviors, and age have all demonstrated important roles in shaping patients’ health behaviors, affecting health outcomes [21,22]. Without acknowledging patients’ priorities and limitations, we cannot provide adequate care; therefore, understanding these issues is crucial for conducting effective medical practice.

## Conclusions

We interviewed a sizeable group of older, low-income, female adults with RA. This population included prior DMARD users and never-users, and they reported experiencing symptoms that could likely be alleviated by DMARDs. Lack of information and/or concerns about side effects were major impediments to DMARD use. Although this population is not representative of all RA patients, it is characterized by a number of attributes (for example, low-income elderly with several comorbidities) that are common in nonadherent RA patient populations. Looking at their responses will help us better understand populations that have been shown to be at high risk for DMARD non-use.

Both non-users and never-users could benefit from increased education at the patient and provider level. Educating primary care providers is important because it would allow them to feel more knowledgeable discussing, recommending, and prescribing DMARDs, which in turn would make patients more comfortable using them. The issue of non-use will likely grow as the gap between supply and demand of providers worsens. Innovative programs to extend the reach of rheumatic disease expertise must be a top priority to improve the use of DMARDs for RA.

## Abbreviations

ACR: American college of rheumatology; DMARD: Disease modifying anti-rheumatic drug; MHAQ: Modified health assessment questionnaire; PACE: Pharmaceutical assistance contract for the elderly; RA: Rheumatoid arthritis; SD: Standard deviation.

## Competing interests

This work was supported by the Arthritis Foundation. Dr. Solomon also receives salary support from the NIH-NIAMS (K24 AR055989 and P60 AR047782).

## Authors’ contributions

EB interpreted the data and drafted the manuscript. KG carried out the interviews, collected the responses, and edited the manuscript. HT performed the statistical analysis and edited the manuscript. DS conceived of the study, drafted its design, assisted with its coordination, and revised the manuscript. Authors all approved the final manuscript.

## Authors’ information

EMB is a research assistant in the Section of Clinical Sciences at Brigham and Women’s Hospital. She holds a BA in Community Health.

KG is a former senior research assistant in the Section of Clinical Sciences at Brigham and Women’s Hospital and current Certified Health Education specialist. She holds an MA in Counseling and Behavioral Medicine and an MS in Health Science.

HT is a Senior Statistical SAS Programmer in the Section of Clinical Sciences at Brigham and Women’s Hospital. He holds an MPH in Biostatistics.

DHS is the Chief of the Section of Clinical Sciences and an attending physician at Brigham and Women’s Hospital. He conducts research in the Division of Rheumatology and Division of Pharmacoepidemiology at the Brigham and is a professor at Harvard Medical School. He holds an MD and an MPH in Epidemiology.
